# A framework for implementing Patient and Public Involvement in mental health research: The PATHWAY research programme benchmarked against NIHR standards

**DOI:** 10.1111/hex.13676

**Published:** 2023-01-10

**Authors:** Lora Capobianco, Cintia Faija, Bethany Cooper, Lindsey Brown, Rebecca McPhillips, Gemma Shields, Adrian Wells

**Affiliations:** ^1^ Research & Innovation, Greater Manchester Mental Health NHS Foundation Trust Manchester Academic Health Science Centre Manchester UK; ^2^ School of Health Sciences, Division of Nursing, Midwifery & Social Work, Faculty of Biology, Medicine and Health University of Manchester Manchester UK; ^3^ Public Contributor Manchester UK; ^4^ School of Health Sciences, Division of Population Health, Health Services Research and Primary Care, Faculty of Biology, Medicine and Health University of Manchester Manchester UK; ^5^ School of Health Sciences, Manchester Centre for Health Economics, Division of Population Health, Health Services Research, and Primary Care, Faculty of Biology, Medicine and Health University of Manchester Manchester UK; ^6^ School of Health Sciences, Center for New treatment and Understanding in Mental Health (CeNTrUM), Division of Psychology and Mental Health, Faculty of Biology, Medicine and Health The University of Manchester Manchester UK

**Keywords:** anxiety, cardiac rehabilitation, depression, metacognitive therapy, Patient and Public Involvement, PPI, psychological therapies

## Abstract

**Background:**

Patient and Public Involvement (PPI) in research has become a key component recommended by research commissioners, grant award bodies and specified in government policies. Despite the increased call for PPI, few studies have demonstrated how to implement PPI within large‐scale research studies.

**Objective:**

The aim of the current study was to provide a case example of the implementation of a patient advisory group in a large‐scale mental health research programme (PATHWAY) and to benchmark this against UK standards.

**Method:**

A PPI group was incorporated throughout the PATHWAY research programme, from grant development to dissemination. The group attended regular meetings and supported participant recruitment, evaluated patient‐facing documents, supported the piloting of the research intervention and co‐developed the dissemination and impact strategy. The implementation of PPI throughout the project was benchmarked against the UK standards for PPI.

**Results:**

The inclusion of PPI in the PATHWAY project provided tangible changes to the research project (i.e., improving study documents, co‐developing dissemination materials) but also proved to be a beneficial experience to PPI members through the development of new skills and the opportunity to provide a patient voice in research. We show how PPI was involved across seven study phases and provide examples of implementation of the six UK standards. The study did not include PPI in data analysis but met all the UK standards for PPI. Challenges regarding practical components (i.e., meeting frequency, language use), increasing diversity and PPI members' knowledge of research were highlighted as areas for further improvement.

**Conclusions:**

We provide a case example of how PPI can be implemented throughout a research lifecycle and we note the barriers faced and make suggestions for PPI in future implementation and research.

**Patient and Public Contribution:**

PPI members were involved throughout the lifecycle of the research programme. The PPI lead was a co‐author on the manuscript and contributed to report writing.

## 1 INTRODUCTION

Patient and Public Involvement (PPI) in research has become prominent in health and social care services research.^1^ PPI activity as defined by the National Institute for Health Research NIHR^2^ is an ‘active partnership between the public and researchers in the research process’ and is demarcated as doing research ‘with’ and ‘by’ the public rather than ‘to’, ‘about’ and ‘for’ the public.

Involving patients and the public in research is strongly recommended by current policy and practice in the United Kingdom,^3^ and funding bodies such as the NIHR and The Research Excellence Framework Higher Education Funding Council emphasize the inclusion of PPI in research programmes.^4^, ^5^, ^6^ In their 2015 report ‘Going the Extra Mile’, the NIHR outlined that by ‘2025 we expect all people using health and social care, and increasing numbers of the public, to be aware of and choosing to contribute to research…’,^6^ ^,p.10^ supporting the public's call for further involvement.

The inclusion of PPI in research has demonstrable benefits such as ensuring that the research conducted is relevant, is culturally and logistically appropriate, enhances recruitment and retention and improves the quality of outputs.^7^, ^8^, ^9^, ^10^ Not only does PPI improve research but it also benefits PPI members. Brett et al.^7^ conducted a systematic review of the impact of PPI on service users, research and communities across 65 studies. Service users reported personal benefits including feeling empowered, valued and making meaningful contributions to the research community. In addition, they reported improved knowledge of research and felt that they were able to further develop skills such as interviewing and public speaking.

While it is evident that PPI has a number of potential benefits to researchers and PPI members, concerns have been raised that it can be tokenistic, managerialist, time consuming and requires significant resourcing.^11^, ^12^ In addition, evidence of the value and impact of PPI remains limited, with inconsistency in what effective PPI in research is, what this might look like^11^ and how to assess impact.^11^ Several systematic reviews have found a poor understanding of how members of the public can be involved and the true impact that PPI can have on research.^7^, ^13^, ^14^, ^15^, ^16^ Such limitations were attributed to the lack of guidance and consistency on how to report and evaluate the use of PPI, which has resulted in the development of tools and guidelines to increase transparency in reporting.^17^, ^18^ More recently, the NIHR^19^ published six UK standards to ensure PPI is effectively implemented, which are (1) inclusive opportunities; (2) working together; (3) support and learning; (4) communications; (5) impact and (6) governance. The standards provide a benchmark for PPI along with indicators that improvement can be monitored against.

While increased guidance on the reporting of PPI in research has been mandated,^5^, ^18^ few studies have provided a case example of how PPI can be effectively implemented throughout the lifecycle of a project. As such, the aims of the present paper are to (1) report on how PPI was utilized in a large‐scale multidisciplinary research programme (PATHWAY) and provide a framework for how PPI can be used and (2) benchmark the PATHWAY trial against the UK standards for PPI.

## 2 OVERVIEW OF THE PATHWAY PROGRAMME

The PATHWAY Programme is a 7‐year project funded under the NIHR Programme Grants for Applied Research (RP‐PG‐1211‐2001). The programme included two feasibility trials and two randomized controlled trials with integrated qualitative and health economics analyses (NCT02420431; NCT03129282; NCT 03999359). The overall aim of the programme was to reduce symptoms of anxiety and/or depression in cardiac rehabilitation (CR) patients by integrating metacognitive therapy (MCT^20^) in CR. PATHWAY evaluated whether MCT delivered in addition to usual CR is more effective than attending usual CR sessions alone in reducing symptoms of anxiety and/or depression. MCT was evaluated in two formats across two different trials, group‐based delivery at CR services (Group‐MCT) and home‐based self‐help with additional telephone support (Home‐MCT) (for details of the programme, see Wells et al.^21^, ^22^, ^23^, ^24^).

## 3 FRAMEWORK FOR PPI: THE PATHWAY TRIAL

**Figure 1:**
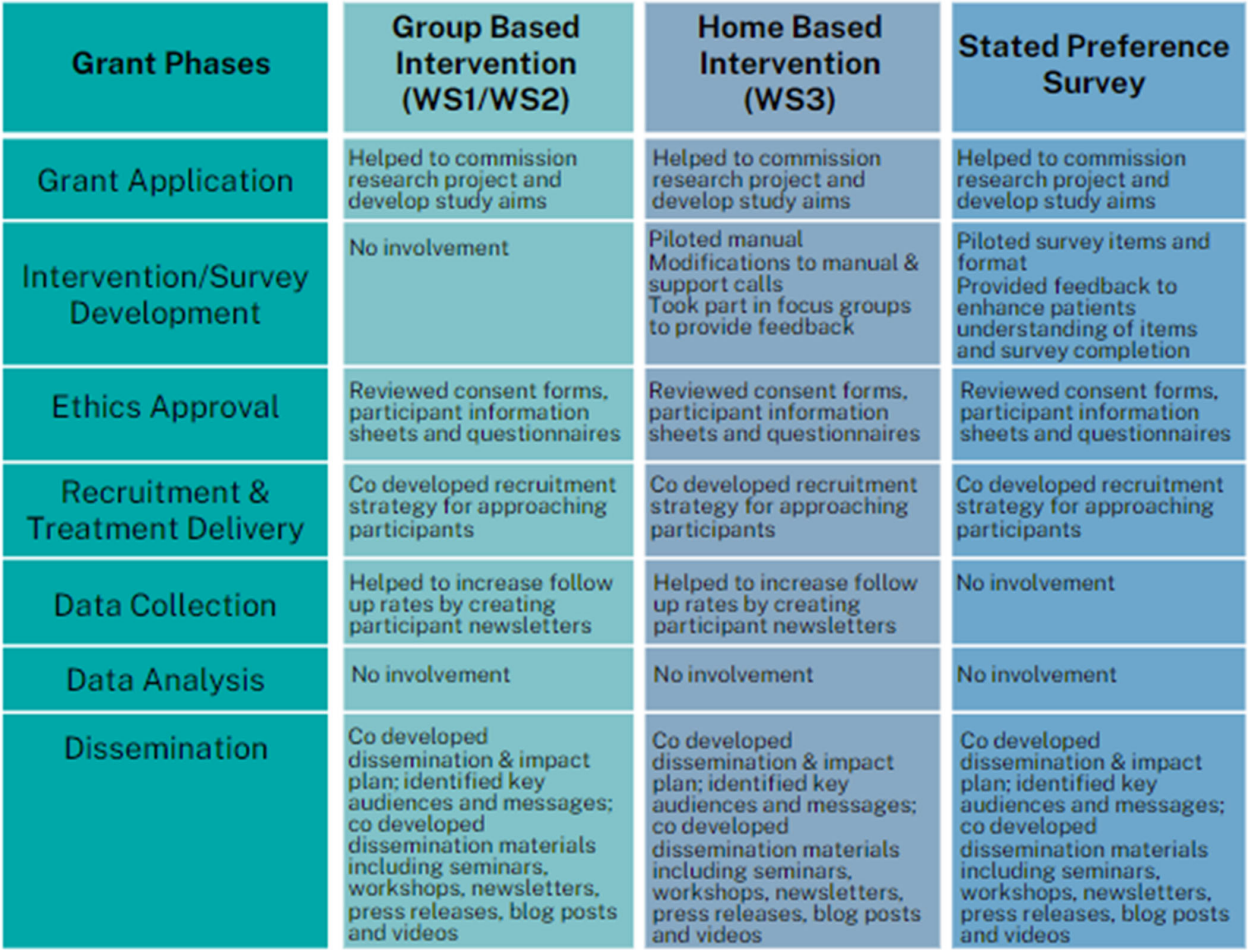
Overview of Patient and Public Involvement throughout all study phases

PPI was integrated throughout all stages of the research programme. Figure 1 presents an overview of the framework for the implementation of PPI. The level of involvement and contribution made by PPI advisors changed throughout the research study and to members' personal preferences. PPI was implemented with the support of a PPI lead who worked in collaboration with both the research team and PPI group. The PPI lead developed and oversaw all aspects of involvement and acted as a facilitator and liaison between both groups, assisted in removing barriers to involvement, acted on concerns raised by the PPI group and evaluated the experiences of the group.

Members of the public were recruited via patient networks, with the following eligibility criteria: experience of one or more of the following was required: (1) heart disease; (2) psychological distress and/or (3) being a carer of someone with one or more of these conditions. A group of 10 advisors was formed following an expression of interest from 16 people. Of the initial 10 advisors, 3 members left. The most common reason for withdrawing was due to illhealth. Demographic characteristics of the 10 PPI advisors and the PPI Lead are included in Table 1. Written consent was sought and obtained from all PPI contributors for their quotes, questionnaire responses and demographic information to be used in this paper.

**Table 1:** Descriptive characteristics of the PPI advisory group

PPI Member ID	Gender	Age at beginning of the study?	Ethnicity	Marital status	Qualification	Are you a CR service user?	Do you have experience in research?	Do you have experience as a PPI member in other research studies?
01	M	Not reported	Not reported	Married	Not reported	Y	N	N
02	F	70	Black African	Divorced	Degree	N (mental health nurse)	Y	Y
03	F	53	Black	Divorced	Degree	Y	Y	Y
04	M	67	White background	Married	Vocational qualification	Y	Y	N
05	M	69	White British	Married	Vocational qualification	Y	N	N
06	M	61	White British	Married	Diploma	Y	N	N
07	F	65	White British	Married	Degree	Y	Y	N
08	F	Not reported	Not reported	Not reported	Not reported	Y (Carer)	Y	Y
09	M	63	White British	Married	Degree	Y	N	N
10^a^	M	Not reported	Not reported	Not reported	Not reported	Not reported	Not reported	Not reported

Abbreviations: F, female; M, male; N, no; PPI, Patient and Public Involvement; Y, yes.

^a^ PPI member left the group before data collection.

### 3.1 Stage 1: Grant development and research governance

Consultations with service users at two cardiac service user group meetings (Service User Research Endeavour Group at Liverpool Heart and Chest Hospital NHS Foundation Trust and the Ticker Club at University Hospital of South Manchester NHS Foundation Trust) were conducted. Two meetings were held, which focused on (1) the research background and preliminary ideas and (2) discussions related to the research proposal and PPI plans. Further consultations, funded by a Research Design Service (RDS) NW bursary, were conducted with Ticker Club members regarding the acceptability and feasibility of the research. An advisor from the RDS NW and a service user were included as co‐applicants on the grant application and reviewed the lay summary and PPI sections of the grant application.

The PPI Lead and the PPI Chair were members of the PATHWAY executive committee, while a PPI member independent of the research team and PPI advisory group was a member of the trial steering committee. As part of the executive and trial steering committee, PPI representatives provided input on study design, conduct, consultation about emerging findings and dissemination and impact planning. Attendance at executive committees allowed the PPI lead and chair to contextualize research findings and review them with the advisory group.

### 3.2 Stage 2: Research delivery

#### 3.2.1 Ethical approval

All patient‐facing material was reviewed and approved by the PPI group before its submission to ethics. This included service users ensuring that the information provided in consent forms and participant information sheets was sufficient and written in a lay format. The advisory group also reviewed the order of study documentation in the information packs to ensure that it was engaging and inviting for prospective patients.

#### 3.2.2 Intervention development

Service users were involved in the development of the home‐based MCT manual, focusing specifically on ensuring that the format and readability of the manual were appropriate for patients.

#### 3.2.3 Home‐based MCT

PPI members contributed to the layout of the home‐based manual. Four PPI members piloted the home‐based manual. In doing so, members completed each module in the manual along with the telephone support calls, which for the purposes of the pilot were delivered by the research team. This allowed researchers to evaluate the timing and content of the telephone support calls. Following the pilot, PPI members provided feedback on the manual and support calls via feedback meetings. PPI members commented that (1) more emphasis should be placed on the ease of use and flexibility in using the modules to enhance compliance; (2) a question section should be added to give the patient the opportunity to clarify/discuss any issues with the therapist in the subsequent telephone call; (3) an estimation of the amount of time to complete each module should be included; (4) the language at times was too technical, which resulted in minor modifications to the text and (5) overall the modules were easy to follow. All of the suggested changes were implemented in the manual ahead of the feasibility trial.

While piloting the home‐based manual benefitted the research team, the advisory group also noted that piloting the self‐help manual supported them in developing further knowledge and understanding of MCT, anxiety and depression. Some advisors felt that piloting the manual changed their beliefs about their own thinking and their personal ways of coping with and understanding anxiety and/or depression symptoms. One PPI member reported that ‘I think it's great that psychological, the emotional side is now being sort of trying to be managed because it's not been there. … I didn′t know until we started getting the paperwork what it was [anxiety and depression] … and now I know why I didn′t want to go out of the house for six months. I never ever suffered with depression before, but I now know why’ (PPI member 3).

#### 3.2.4 Recruitment and treatment delivery

The PPI group also aided in co‐developing a recruitment strategy for approaching participants across all workstreams. This ensured that the language used when approaching and inviting potential participants to take part was appropriate and engaging. In addition, for the health economics workstream, the group provided feedback on potential sources of recruitment.

#### 3.2.5 Data collection and analysis

To increase the rate of return of follow‐up questionnaires, service users were asked for suggestions on how to enhance follow‐up participation and decrease the drop‐out rate. Suggestions included the creation of a participant newsletter, which highlighted the importance of questionnaire completion and its benefit to patients and NHS services while providing trial updates and maintaining patient engagement. The newsletter was co‐produced between PPI members and the research team and provided a significant benefit in improving the rate of return for follow‐up questionnaires.

Service users were not involved in the data analysis, although qualitative and quantitative data were discussed with the PPI group, which aided in ensuring that key messages were presented in a format that was accessible to the public; for example, service users commented on the importance of including the treatment recovery rate, as they felt this was an important outcome for patients.

#### 3.2.6 Stated preference research

A stated preference survey using a discrete choice experiment was designed to estimate preferences for psychological care within the CR pathway.^25^ PPI members advised on the design of the discrete choice experiment, including feedback on potential key attributes and levels to be used in choice set questions, refining these to create a feasible design and feedback on participant demographics that may affect preferences for care. The PPI group also completed and commented on a draft survey to check that materials were easy to follow and to estimate the time that would be required by participants. Finally, the group commented on the likely impact of the COVID‐19 pandemic on stated preference responses (including the response rate and elicited preferences). The PPI work was published as a case study focusing on the use of PPI in stated preference research in health.^26^ The PPI lead (L. B.) was an author of the case study and contributed to report writing.

#### 3.2.7 Qualitative process evaluation

A qualitative process evaluation was embedded into the PATHWAY trial. The PPI group was consulted about the design of the qualitative process evaluation and the materials used to collect qualitative data. Initially, the group provided feedback on the longitudinal nature of the qualitative component of the research and confirmed that they felt this would not be burdensome for participants. The group was also consulted about the qualitative interview topic guides, with the aim of ensuring that all areas of relevance were covered during interviews, the correct language was used and topics that could be understood to be potentially sensitive were handled in an appropriate way.

### 3.3 Stage 3: Dissemination

Group activities were held with the PPI group to aid the development of the dissemination plan with a focus on (1) identifying key messages, target audiences, format and publication/venues and (2) styles of dissemination. Discussions were conducted to determine results of relevance to the public, explanations of future steps and language use in dissemination work. The group aided in the development of resources with members being involved in creating videos, blogs and presentations of their experiences taking part in PPI, which helped to provide a patient voice. They also played a key role in the development and delivery of a live event disseminating the study results for patients, clinicians and members of the public: helping to inform the format of the event and taking part in the event by discussing why they chose to be involved in the project and the role they played in the research.

#### 3.3.1 PPI group feedback

**Figure 2:**
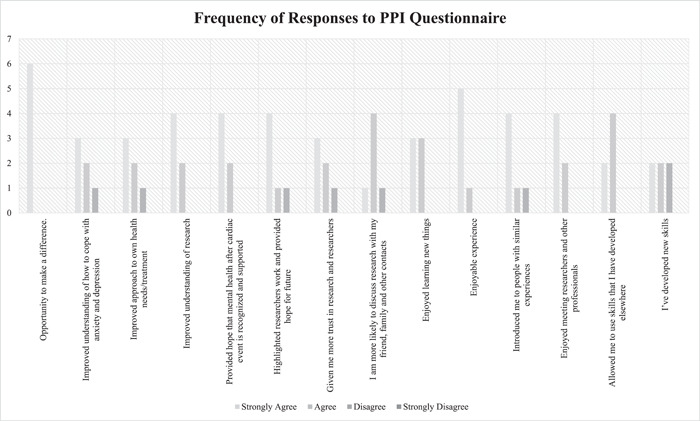
Frequency of PPI responses to PPI Questionnaire. PPI, Patient and Public Involvement.

Evaluations of the PPI approach were conducted throughout the project through feedback meetings, telephone calls and a summative questionnaire completion (see Supporting Information: Appendix 1) by the PPI lead. The summative questionnaire was developed by the study team to assess the impact that taking part in a PPI group has had on them. These evaluations encouraged members to raise concerns, highlight the benefits of participating in research and determine how the group met NIHR standards. The summative questionnaire was completed in the final year of the study. Results were analysed and anonymized by the PPI lead before being shared with the PPI and research teams. Six members completed the questionnaire. Overall, PPI members felt that taking part provided them with the opportunity to make a difference, improved their understanding of research, made them more likely to discuss research with friends and family and improved their understanding of coping with anxiety and depression and how they personally manage it (Figure 2). Only one PPI member felt that taking part in the PPI group did not improve their understanding of anxiety and depression, their own mental health coping or their perspectives on researchers. This was due to their occupation and previous research experience. All PPI members noted that taking part did not have any negative impacts on them and four out of six would take part in further PPI. The two members who said they would not take part in further PPI noted that this was due to a deterioration in their physical health.

## 4 BENCHMARKING PPI

To assess the implementation of PPI in PATHWAY, we compared our use against the NIHR UK standards (see Table 2). In addition to benchmarking against UK standards, we note the challenges faced and ways in which the standards could have been further implemented. Feedback meetings were held with the PPI group to assess how they felt the team met the NIHR UK standards. See Table 3 for a summary of key recommendations for future PPI groups.

**Table 2:** Implementation of NIHR UK standards

Indicators	Research phase	PATHWAY implementation
1. Inclusive opportunities	GA	Recruited a range of public contributors (patients and carers) from varying sources (i.e., cardiovascular charities, public involvement groups)
	GA	Removed barriers by asking about any support needs from the first meeting and ensured this was supported throughout
	All	Providing financial support for their time and travel
	All	PPI members were given the choice for how they would like to be involved based on members' interest and/or skills
2. Working together	All	Collaborative approach from small tasks (i.e., meeting dates) to co‐productive of materials and dissemination activities
	All	Decisions made collaboratively with researchers
	GA	Created terms of reference with the PPI group, which was reviewed quarterly
	All	Ongoing review of roles and expectations
	All	Individual members' ideas and contributions recognized, felt valued and listened to
	All	Meeting minutes circulated following each meeting
	All	Open dialogue and communication
3. Support and learning	All	Clear point of contact for information and support, always accessible to answer questions
	All	Supported individual's needs (i.e., visual disabilities)
	All	Designated resources to support public involvement
	All	Informing the group about PPI activities external to the project, especially as the project ends
	D	Learning by doing (co‐presenting with researchers)
4. Communications	All	Flexibility in providing feedback to members following meetings (via email or phone)
	All	Feedback provided at meetings on how the research team has implemented feedback or justification for why feedback could not be implemented was provided at each meeting.
	D	Co‐developed a dissemination and impact plan
5. Impact	D	Co‐developed a dissemination and impact plan
6. Governance	All	Service users involved throughout all phases of the study
	All	Service user representation on the executive committee and steering committees
	All	Money and resources allocated for public involvement
	All	Terms of reference reviewed quarterly

Abbreviations: All, all phases; D, Dissemination; GA, grant application; PPI, Patient and Public Involvement.

**Table 3:** Key recommendations for future PPI groups

UK standard	Recommendations
1. Inclusive opportunities	Ensure PPI is adequately costed for, tools such as the INVOLVE calculator can aid with financing
	Ensure a range of PPI members are recruited using various ways of advertising (online, local groups) and allow flexibility in ways of returning an expression of interest forms
	Provide PPI members with flexibility and choice in how they would like to provide feedback (i.e., in meetings, phone, via email) and when (i.e., more/less contribution at different phases)
2. Working together	Create a term of reference document at the outset that includes aspects such as frequency of meetings, times, amount of notice required before a meeting is to be scheduled, to aid in setting expectations
	Ensure a collaborative approach and co‐production of materials and dissemination and impact planning
3. Support and learning	Ensure groups have a clear point of contact (i.e., designated researcher or PPI lead)
	Ensure individual's needs are met (i.e., visual disabilities)
	Ask PPI members if they would be interested in learning about other PPI opportunities to support their engagement in the PPI group
	Allow members to co‐present with researchers to aid in developing new skills
4. Communications	Ensure feedback is provided to outline why suggestions made by the PPI group have or have not been implemented
5. Impact	Involve PPI members in developing the research dissemination and impact plan (identifying who to disseminate to, selecting key messages for varying audiences)
6. Governance	Involve PPI throughout all governance levels

Abbreviation: PPI, Patient and Public Involvement.

### 4.1 Standard 1: Inclusive opportunities

Feedback from the PPI group highlighted that they felt inclusive opportunities were met early on with the adaptations made to support the needs of individual members and provide choice and flexibility around the type of work the group would be involved in. All group members were reimbursed for their time following UK standards along with travel expenses, to avoid financial barriers to involvement. Considerations raised for future involvement highlighted that members may have disabilities that make taking part in group settings difficult and therefore one‐to‐one options could have been provided for involvement. While the diversity of the PPI group is representative of those patients recruited to the PATHWAY trial, recruitment to the PPI group could have utilized a broader recruitment strategy. Participants were predominantly recruited via local patient networks or online advertisement; however, future research could aim to recruit directly from the patient services they aim to conduct research with.

### 4.2 Standard 2: Working together

Feedback meetings, telephone calls and questionnaires were used throughout the project, to evaluate the PPI approach and allow challenges to be raised and addressed to improve working together and was led by the PPI lead. This was met positively by the PPI group as it presented an opportunity for them to talk anonymously away from the research team. The phone calls in particular were considered to offer an opportunity to raise concerns that would not normally be raised in a larger group. Feedback from evaluations resulted in changes to our PPI approach. For example, the room layout was changed from a classroom style to a round table, more time was allocated to discussions rather than presentations and more clarity was given about which areas of the research could not be changed and why. In addition, at the start of meetings, we implemented a ‘you suggested, we did’, where we reviewed changes that we were able to implement based on discussions from the previous meeting. When changes were unable to be implemented due to research processes, these were clearly explained to the PPI group. Small actions such as using doodle polls and introducing all new members of the team were also felt to have played a role in creating an atmosphere of equality and clarity between the PPI and research teams. By the end of the study, our approach to working together was felt to strength, as one member said, ‘It has been a fulfilling experience. Too often patients' views are not considered I felt that we were listened to at every stage of the research and our suggestions were treated with respect’ (PPI Member 7).

In the future, the group commented that further assistance was needed for individuals reintegrating into the group when they had been away for personal or health reasons. This could have been facilitated by a one‐to‐one meeting with the PPI lead and PPI member before the meeting to update on any changes to the group and progress made during missed meetings.

### 4.3 Standard 3: Support and learning

Advisory group members felt well supported; measures were put in place to accommodate those with additional needs or disabilities from the start of the project and were reviewed regularly. In addition, there was a range of learning opportunities offered to the group throughout the project, such as working with the team on presentations. PPI members also communicated that taking part in PPI had increased their trust in research and researchers and made them more likely to discuss research with friends and family. PPI members commented on the increased understanding they gained, ‘I have gained a better understanding of all the difficult decisions that researchers have to make’ (PPI Member 5). There were however a number of ways in which learning opportunities could have been further supported. While the advisory group ultimately felt that sufficient information had been given, at times confusion around some of the processes, protocols and unfamiliarity with the psychological intervention (MCT) had resulted in feelings of being under‐utilized. For example, although the psychological intervention used within the trial (MCT) was explained to the group, this was not done until later in the research programme. One member commented that,

when we first started most, possibly all of us didn′t understand the nature of the actual treatment. It was quite a long way down the line before we saw the materials … and understood more about how the treatment would work. I think some training earlier on in the process would have put us in a position where we were better informed to contribute. (PPI Member 6)

The group also felt that, while the research team provided sufficient information on the research process, this was a high volume of information, and providing them with a handbook or online training materials outlining the research processes would have been useful: allowing them to look up aspects of the research and refresh on processes in their own time. Providing such information at the start of the research process could have also aided in setting expectations with the PPI team around what they could and could not change. For example, PPI members wondered if it was possible to change the wording on validated questionnaires, which was outside the scope of what they were able to do. However, the research team did provide an explanation as to why this was not possible, the implications for the project and how questionnaires are developed.

Throughout the project, we had PPI representation at both the steering and executive team meetings and the principal investigator met with the PPI lead at these meetings and also at the PPI groups. To support PPI members and to ensure they felt comfortable attending these meetings, the PPI lead would accompany the PPI member to the meetings. PPI members were also provided with a copy of the previous meeting minutes, the meeting slides and the meeting agenda before the meeting. Following the meeting, the PPI lead and the research fellow were available for the PPI member to contact and ask questions if they felt uncomfortable to asking these during the team meeting.

### 4.4 Standard 4: Communication

The PPI group felt that the varying types of communication (group‐based, one‐to‐one, emails, phone calls) and amount of communication outside of meetings were sufficient. However, the initial frequency of meetings, format and approach of meetings required improvements. While recaps of previous meetings were originally provided at the start of each meeting to reduce the impact of long gaps between meetings, it was viewed by members as wasting time and detracting from project business, as such email communication between meetings was increased to reduce the length of the recap at subsequent meetings. The group felt that they were able to speak without opinions being dismissed and that this also allowed them to say when they did not understand. They felt the research team was responsive and adjusted their language (i.e., used plain language) when speaking with PPI members. However, one member highlighted that expressing a different opinion to the rest of the group can be intimidating and can result in the feeling that they have to agree with the consensus. As such, members were specifically encouraged to express alternative views, and varying formats were offered to provide feedback (i.e., one‐to‐one, emails, phone calls), which is paramount in ensuring open and honest feedback from members.

### 4.5 Standard 5: Impact

The advisory group felt that their input was taken on board and the research team was very open to feedback from them. Being involved in the co‐development of the home‐based MCT intervention was felt by the group to be their most significant impact as they could see the adaptions following their suggestions. Advisors felt their input on this also had the most impact on patients as it ensured the accessibility of the home‐based intervention.

### 4.6 Standard 6: Governance

The structure of governance included a PPI lead and PPI chairman as members of the executive committee and an independent PPI member as a member of the steering committee. This helped to ensure that PPI representation was considered at all project governance levels. Reviews of the PPI approach were conducted throughout the project and adjustments were made where needed. Future considerations were suggestions surrounding funding for PPI activities, as there were some additional opportunities that the group was unable to undertake due to funding not being allocated for them. Therefore, future planning for public involvement should include the allocation of funding for additional PPI opportunities such as attendance at conferences. Further suggestions include clarification of what payments can cover (i.e., taxis to attend meetings) and ensuring clear procedures around paperwork and timing of payments to ensure clear expectations on how long a payment may take.

## 5 DISCUSSION

PPI can play an important role by providing a patient's perspective that can be utilized throughout all phases of a research study. The current paper illustrated how PPI can be implemented as well as how national PPI standards might be met, and implementation improved upon further.

### 5.1 Implementation

Throughout the research programme, PPI resulted in tangible changes in patient‐facing documents, the layout of the home‐based intervention, the development of newsletters and participation in dissemination events. In doing so, PPI members aided in ensuring the appropriateness of the language being used, the format of interventions and aided in ensuring research findings were presented in an accessible and user‐friendly manner. The implementation of PPI in our PATHWAY programme was consistent with previous studies, which have advocated for PPI throughout all phases of research.^12^, ^19^, ^27^ Beyond playing an integral role in the research programme, PPI also provided benefits to PPI members, an often‐overlooked benefit to implementing PPI in research. In line with previous research,^12^, ^27^, ^28^ PPI members noted that the study allowed them to develop new transferrable skills, allowed them to have a voice, connect with others who had similar experiences and connect the study results to the impact and benefit for patients.

### 5.2 Benchmarking and future learning

To assess if PPI met national guidelines for quality and consistency, we benchmarked our strategy against the UK standards for public involvement.^19^ While some authors have argued that the UK standards could lead to inflexible PPI practices,^29^ the standards allowed us to reflect on the way PPI was utilized and highlighted ways in which it might have been improved.^30^ Conducted a systematic review of PPI frameworks, which found that there were 65 different PPI frameworks that could be grouped into five categories: power‐focused; priority‐setting; study‐focused; report‐focused and partnership‐focused. While this highlights that there are several frameworks available with specific objectives to help guide PPI. But specificity may compromise transferability, and^30^ note little use beyond the groups that originally developed them. Guidelines that are broader and provide principles to work towards such as INVOLVE may provide more flexibility and stimulate use across objectives.

One of the main sources of difficulty that we observed between researchers and advisory group members was the advisory groups' lack of knowledge of the research processes, which is commonly noted as a barrier.^12^, ^27^, ^31^ In the future, we recommend that researchers should hold a training event at the study's outset, which focuses on research processes/timelines, confidentiality and what can happen at the end of a research project. This should assist in setting realistic expectations for what the research can achieve within a limited time frame and what is within the scope of the research project. For example, in research projects evaluating an intervention that is found to be effective, it would be useful to provide an outline of the duration and the steps that are needed to be undertaken before new treatments become accessible to the public. We found that the PPI group held unrealistic expectations concerning the implementation of research findings. Additionally, studies may also look to provide a research handbook for PPI groups that members can use to refer back to throughout the project and in their own time.^32^

In addition, there were practical challenges that were encountered in implementing PPI including frequency of meetings, format and delivery of meetings and language use. At times, there were long gaps between meetings, which created inconsistency and feelings of underutilization. In the future, setting clear expectations at the study's outset can aid in establishing accountability between researchers and the PPI group. According to Ocloo et al.'s^28^ systematic review of the barriers and enablers of PPI across health and social care research, clear definitions of roles, goals of participation and specification of the time commitment required were important factors in improving PPI.

While a common challenge of PPI surrounds issues of power dynamics,^12^, ^27^, ^28^, ^29^, ^30^, ^31^ the PATHWAY programme did not (to our knowledge) encounter observable difficulties in this area; however, we are mindful that such dynamics might exert unseen influences. Our interpretation of the lack of observable problems in this domain is that PPI was included at programme governance levels (steering committee, executive team committees) and a designated PPI lead acted as a bridge between the PPI group and researchers. We also incorporated PPI throughout the project and solicited ongoing reflections on PPI utilization that was facilitated by the PPI lead. Such strategies may have helped to reduce the ‘us vs. them’ culture often noted as a barrier in implementing PPI. However, further assessments of the mechanisms that may mitigate such dynamics are required.

### 5.3 Limitations

While the PATHWAY programme incorporated PPI throughout the lifecycle of the research project, further assessment of the impact and effectiveness of PPI in PATHWAY is required. Assessment of PPI was conducted using a questionnaire developed by the study team, but this measure has not been validated and items focused largely on assessing the impact of taking part in a PPI group. It would have been useful to include assessments of the barriers and enablers to PPI engagement during sessions and to examine the personal frameworks used by PPI members in assessing and advising on the research process. Similarly, an evaluation was not conducted on the impact that PPI had on the researchers themselves.^12^ evaluated researchers' experiences and perceptions of PPI in 36 health researchers and noted the complexity of researchers' experience in involving patients and the public in research. While researchers noted a range of practical challenges (i.e., lack of time, insufficient funding), they also noted that PPI was accompanied by a range of emotions from cynicism to enthusiasm, noting that the impact that PPI has on research is both beneficial and burdensome.

## 6 CONCLUSION

The current paper provided an account of how PPI was implemented throughout the lifecycle of PATHWAY. PPI was a component of our clinical trials, stated preference survey and qualitative research. We benchmarked PPI against UK standards, and although we appear to be compliant, we found specific areas for improvement. We provide an overview and examples of our practice in the PATHWAY programme and make specific suggestions that could be useful in guiding future studies.

## AUTHOR CONTRIBUTIONS

Lora Capobianco and Adrian Wells contributed to conceptualization, investigation, writing – original and subsequent drafts and editing and project administration. Cintia Faija, Bethany Cooper, Lindsey Brown, Rebecca McPhillips and Gemma Shields contributed to the writing – review and editing. Adrian Wells contributed to supervision and funding acquisition.

## CONFLICT OF INTEREST

A. W. is the director of the MCT Institute and developer of MCT. A. W. has written books on cognitive behaviour therapy and MCT. The remaining authors declare no conflict of interest.

## Supporting information

Supporting information.

## Data Availability

Data may be made available on request to the corresponding author.

## References

[1] Gibson A , Britten N , Lynch J . Theoretical directions for an emancipatory concept of patient and public involvement. Health (London). 2012;16(5):531‐547. 22535648 10.1177/1363459312438563

[2] NIHR. Glossary NIHR [online]. National Institute for Health Research; 2021. https://www.nihr.ac.uk/glossary?letter=P&postcategory=-1

[3] Department of Health. Research Governance Framework for Health and Social Care. 2005—Annex. Second edition. Department of Health; 2008. https://assets.publishing.service.gov.uk/government/uploads/system/uploads/attachment_data/file/139565/dh_4122427.pdf

[4] INVOLVE . Briefing Notes for Researchers: Involving the Public in NHS, Public Health and Social Care Research. INVOLVE; 2012. https://www.invo.org.uk/wp-content/uploads/2012/04/INVOLVEBriefingNotesApr2012.pdf

[5] NIHR. Briefing Notes for Researchers—Public Involvement in NHS, Health and Social Care Research. National Institute for Health Research; 2021. https://www.nihr.ac.uk/documents/briefing-notes-for-researchers-public-involvement-in-nhs-health-and-social-care-research/27371

[6] NIHR. Going the Extra Mile: Improving the Nation's Health and Wellbeing Through Public Involvement in Research. National Institute for Health Research; 2015. https://www.nihr.ac.uk/documents/about-us/our-contribution-to-research/how-we-involve-patients-carers-and-the-public/Going-the-Extra-Mile.pdf

[7] Brett J , Staniszewska S , Mockford C , et al. A systematic review of the impact of patient and public involvement on service users, researchers and communities. Patient. 2014;7(4):387‐395. 10.1007/s40271-014-0065-0 25034612

[8] Crocker JC , Ricci‐Cabello I , Parker A , et al. Impact of patient and public involvement on enrolment and retention in clinical trials: systematic review and meta‐analysis. BMJ. 2018;363:k4738. 10.1136/bmj.k4738 30487232 PMC6259046

[9] Jagosh J , Macaulay AC , Pluye P , et al. Uncovering the benefits of participatory research: implications of a realist review for health research and practice. Milbank Q. 2012;90(2):311‐346. 10.1111/j.1468-0009.2012.00665.x 22709390 PMC3460206

[10] Staley K . Exploring Impact: Public Involvement in NHS, Public Health and Social Care Research. INVOLVE; 2009. https://www.invo.org.uk/wp-content/uploads/2011/11/Involve_Exploring_Impactfinal28.10.09.pdf

[11] Madden M , Speed E . Beware zombies and unicorns: toward critical patient and public involvement in health research in a neoliberal context. Front Sociol. 2017;2:7. 10.3389/fsoc.2017.00007

[12] Boylan AM , Locock L , Thomson R , Staniszewska S . “About sixty per cent I want to do it”: health researchers' attitudes to, and experiences of, patient and public involvement (PPI)—a qualitative interview study. Health Expect. 2019;22(4):721‐730. 10.1111/hex.12883 30927334 PMC6737750

[13] Brett J , Staniszewska S , Mockford C , et al. Mapping the impact of patient and public involvement on health and social care research: a systematic review. Health Expect. 2012;17(5):637‐650. 22809132 10.1111/j.1369-7625.2012.00795.x PMC5060910

[14] Crawford MJ . Systematic review of involving patients in the planning and development of health care. BMJ. 2002;325(7375):1263. 12458240 10.1136/bmj.325.7375.1263 PMC136920

[15] Mockford C , Staniszewska S , Griffiths F , Herron‐Marx S . The impact of patient and public involvement on UK NHS health care: a systematic review. Int J Qual Health Care. 2011;24(1):28‐38. 22109631 10.1093/intqhc/mzr066

[16] Smith E , Ross F , Donovan S , et al. Service user involvement in nursing, midwifery and health visiting research: a review of evidence and practice. Int J Nurs Stud. 2008;45(2):298‐315. 17161402 10.1016/j.ijnurstu.2006.09.010

[17] Staniszewska S , Brett J , Mockford C , Barber R . The GRIPP checklist: strengthening the quality of patient and public involvement reporting in research. Int J Technol Assess Health Care. 2011;27(4):391‐399. 10.1017/S0266462311000481 22004782

[18] Staniszewska S , Brett J , Simera I , et al. GRIPP2 reporting checklists: tools to improve reporting of patient and public involvement in research. BMJ. 2017;358:j3453. 10.1136/bmj.j3453 28768629 PMC5539518

[19] NIHR. National Standards for Public Involvement. National Institute for Health Research; 2019. https://sites.google.com/nihr.ac.uk/pi-standards/standards

[20] Wells A . Metacognitive Therapy for Anxiety and Depression. Guilford Press; 2009.

[21] Wells A , McNicol K , Reeves D , et al. Improving the effectiveness of psychological interventions for depression and anxiety in the cardiac rehabilitation pathway using group‐based metacognitive therapy (PATHWAY Group MCT): study protocol for a randomised controlled trial. Trials. 2018;19(1):215. 29615092 10.1186/s13063-018-2593-8 PMC5883514

[22] Wells A , McNicol K , Reeves D , et al. Metacognitive therapy home‐based self‐help for cardiac rehabilitation patients experiencing anxiety and depressive symptoms: study protocol for a feasibility randomised controlled trial (PATHWAY Home‐MCT). Trials. 2018;19(1):444. 30115112 10.1186/s13063-018-2826-x PMC6097432

[23] Wells A , Reeves D , Heal C , et al. Establishing the feasibility of group metacognitive therapy for anxiety and depression in cardiac rehabilitation: a single‐blind randomized pilot study. Front Psychiatry. 2020;11:582. 32714216 10.3389/fpsyt.2020.00582 PMC7344162

[24] Wells A , Reeves D , Capobianco L , et al. Improving the effectiveness of psychological interventions for depression and anxiety in cardiac rehabilitation: PATHWAY—a single‐blind, parallel, randomized, controlled trial of group metacognitive therapy. Circulation. 2021;144(1):23‐33. 34148379 10.1161/CIRCULATIONAHA.120.052428 PMC8247550

[25] Shields GE , Wright S , Wells A , Doherty P , Capobianco L , Davies LM . Delivery preferences for psychological intervention in cardiac rehabilitation: a pilot discrete choice experiment. Open Heart. 2021;8(2):e001747. 10.1136/openhrt-2021-001747 34426529 PMC8383873

[26] Shields GE , Brown L , Wells A , Capobianco L , Vass C . Utilising patient and public involvement in stated preference research in health: learning from the existing literature and a case study. Patient. 2021;14(4):399‐412. 10.1007/s40271-020-00439-2 32748242 PMC8205869

[27] The PARTNERS2 Writing Collective . Exploring patient and public involvement (PPI) and co‐production approaches in mental health research: learning from the PARTNERS2 research programme. Res Involv Engagem. 2020;6:56. 10.1186/s40900-020-00224-3 32974052 PMC7507647

[28] Ocloo J , Garfield S , Franklin BD , Dawson S . Exploring the theory, barriers and enablers for patient and public involvement across health, social care and patient safety: a systematic review of reviews. Health Res Policy Syst. 2021;19:8. 10.1186/s12961-020-00644-3 33472647 PMC7816359

[29] McCoy MS , Jongsma KR , Friesen P , et al. National standards for public involvement in research: missing the forest for the trees. J Med Ethics. 2018;44:801‐804. 30337451 10.1136/medethics-2018-105088

[30] Greenhalgh T , Hinton L , Finlay T , et al. Frameworks for supporting patient and public involvement in research: systematic review and co-design pilot. Health Expect. 2019;22(4):785‐801. 10.1111/hex.12888 31012259 PMC6737756

[31] Ocloo J , Matthews R . From tokenism to empowerment: progressing patient and public involvement in healthcare improvement. BMJ Qual Saf. 2016;25(8):626‐632. 10.1136/bmjqs-2015-004839 PMC4975844 26993640

[32] Bee P , Brooks H , Callaghan P , Lovell K , eds. A Research Handbook for Patient and Public Involvement Researchers. Manchester University Press; 2018. 10.7765/9781526136527

