# Supplementary material for: A framework for implementing Patient and Public Involvement in mental health research: The PATHWAY research programme benchmarked against NIHR standards
Source: Health Expect. 2023 Jan 10;26(2):640–50. doi: 10.1111/hex.13676 (PMC10010097; doi:10.1111/hex.13676)
Supplement: Supplementary file 1 — Supporting information. [file HEX-26--s001.docx]

**Appendix A. PPI Questionnaire**

**1. Had you been involved in research before joining the MCT PATHWAY Advisory group (i.e. working with researchers rather than being a research participant)?**

Yes

No

**2. Have been involved in any new research projects since joining the MCT PATHWAY Advisory group (working with researchers rather than being a research participant)?**

Yes

No

**3. We would like to know in what ways being a public contributor on this project has had a positive impact on you. Please rate the following statements.**

|  | Strongly Disagree | Disagree | Agree | Strongly Agree |
| --- | --- | --- | --- | --- |
| Given me an opportunity to make a difference. | 1 | 2 | 3 | 4 |
| Improved my understanding of how to cope with anxiety and depression | 1 | 2 | 3 | 4 |
| Improved my approach to my own health needs and/or treatment | 1 | 2 | 3 | 4 |
| Given me a better understanding of how research works | 1 | 2 | 3 | 4 |
| Given me more hope that anxiety and depression following a cardiac event has been recognised and that more people will be supported in the future | 1 | 2 | 3 | 4 |
| Shown me that researchers are working to find treatments for a wide range of conditions and it has made me feel more hopeful for the future. | 1 | 2 | 3 | 4 |
| Given me more trust in research and researchers | 1 | 2 | 3 | 4 |
| I am more likely to discuss research with my friend, family and other contacts | 1 | 2 | 3 | 4 |
| Enjoyed learning new things | 1 | 2 | 3 | 4 |
| Enjoyable experience | 1 | 2 | 3 | 4 |
| Introduced me to people who have experienced similar things to me | 1 | 2 | 3 | 4 |
| Enjoyed meeting researchers and other professionals | 1 | 2 | 3 | 4 |
| Allowed me to use skills that I have developed elsewhere | 1 | 2 | 3 | 4 |
| I’ve developed new skills | 1 | 2 | 3 | 4 |

**4 Are there any comments that you would like to make to clarify your answers above?**

**5.Are there any other comments that you would like to make about how being involved in this project has had a positive effect on you or about your experience of being involved in this project?**

**6. Has being a public contributor on this project had any negative impacts on you?**

Yes

No

If ‘Yes’ please tell us about these:

**7. Would you like to take part in involvement (working with researchers rather than being a research participant) again?**

Yes

No
